# Development of a Weighted-Incidence Syndromic Combination Antibiogram (WISCA) to guide the choice of the empiric antibiotic treatment for urinary tract infection in paediatric patients: a Bayesian approach

**DOI:** 10.1186/s13756-021-00939-2

**Published:** 2021-05-01

**Authors:** Elisa Barbieri, Daniele Bottigliengo, Matteo Tellini, Chiara Minotti, Mara Marchiori, Paola Cavicchioli, Dario Gregori, Carlo Giaquinto, Liviana Da Dalt, Daniele Donà

**Affiliations:** 1 Division of Paediatric Infectious Diseases- Department of Women’s and Children’s Health, University of Padova, Padova, Italy; 2 Department of Cardiac, Thoracic and Vascular Sciences, Unit of Biostatistics, Epidemiology and Public Health, University of Padova, Padova, Italy; 3 Paediatric Emergency Unit - Department of Women’s and Children’s Health, University of Padova, Padova, Italy; 4 Department of Paediatrics, Ospedale Dell’Angelo, Mestre, Venice, Italy

**Keywords:** Urinary tract infection, Combination antibiogram, Children, Bayesian model, Antibiotic-resistance, Antimicrobial stewardship

## Abstract

**Background:**

To evaluate the ability of Weighted-Incidence Syndromic Combination Antibiograms (WISCA) to inform the selection of empirical antibiotic regimens for suspected paediatric community-acquired urinary tract infections.

**Methods:**

Data were collected from outpatients (< 15 years) accessing the emergency rooms of Padua University-Hospital and Mestre Dell' Angelo-Hospital (Venice) between January 1st, 2016, and December 31st, 2018. WISCAs were developed by estimating the coverage of eight regimens using a Bayesian hierarchical model adjusted for age, sex, and previous antibiotic treatment or renal/urological comorbidities.

**Results:**

385 of 620 urine culture requests were included in the model analysis. The most frequently observed bacterium was *E. coli* (85% and 87%, Centre A and B). No centre effect on coverage estimates was found, and data were successfully pooled together. Coverage ranged from 77.8% (Co-trimoxazole) to 97.6% (Carbapenems). Complex cases and males had significantly lower odds of being covered by a regimen than non-complex cases and females (odds ratio (OR) 0.49 [95% HDI, 0.38–0.65], and OR: 0.73 [95% HDIs, 0.56–0.96] respectively). Children aged 3–5 years had lower odds of being covered by a regimen than other age groups, except for neonates.

**Conclusions:**

The developed WISCAs provide highly informative estimates on coverage patterns overcoming the limitation of combination antibiograms and expanding the framework of previous Bayesian WISCA algorithm.

**Supplementary Information:**

The online version contains supplementary material available at 10.1186/s13756-021-00939-2.

## Background

Urinary tract infections affect 2–5% of children, and the incidence varies significantly according to patients' age, sex, ethnicity, presence of circumcision and/or genitourinary malformation, and immune system [1–3]. The etiology is often bacterial, and the three most frequently involved pathogens are *E. coli*, *Klebsiella* spp. and *P. mirabilis*.[3]

The treatment strategy choice should be made promptly since several complications can arise, such as the destruction of tissues, scar tissue formation, sepsis, and, although rarely, death. [4, 5] When prescribing an empirical antibiotic treatment, different factors should be taken into account, including the increase in rates of multi-drug resistance organisms (MDRO) worldwide that pose a threat to patient safety, and the fact that overprescribing of antibiotics increases the selection of resistant bacteria strains.

To solve this conundrum, the so-called combination antibiogram [6] has been developed to support the clinician to make a more informed decision in the selection of empirical antimicrobial therapy by estimating that at least one drug will act on a given pathogen, reducing the culture results' waiting time. However, combination antibiogram is not disease-specific and cannot be used at a population level due to differences in bacteria prevalence rates and the known differences within different age groups [7].

In response to these limitations, Hebert et al. [8] developed the WISCA (Weighted-Incidence Syndromic Combination Antibiogram), a tool that estimates the likelihood that each antibiotic regimen will treat all relevant organisms for a given infection syndrome based on the frequency of the causative pathogen sensitivity. In contrast to the combination antibiogram, less frequent pathogens have less weight on the overall coverage estimate for the same infection syndrome.

The construction of the WISCA for community acquired urinary tract infection (CA-UTI) is the first step toward its use as a tool in supporting antimicrobial stewardship policy in paediatrics (i.e., development of a clinical pathways-based stewardship), as it provides more accurate estimates on coverage patterns, overcoming the limitations of the combination antibiograms.

A study by Randhawa et al. [9] found that WISCA had the potential to more than double the likelihood of adequate empiric antibiotic coverage among patients admitted to the intensive care unit with ventilator-associated pneumonia and catheter-associated bloodstream infection. Moreover, a recent randomized controlled trial on a WISCA antibiotic stewardship clinical decision support tool conducted in the US, found that providers in the intervention group followed recommendations to change antibiotics 60% of the time. [10]

As previously noted in other studies, [11–13] there are still analytical challenges in the WISCA development, represented mainly by the paucity of data in the different strata in the models that can be overcome using Bayesian methods.

This study aims to develop a stratified WISCA to define the most appropriate empiric treatment in children with CA-UTIs.

## Methods

### Study design and population

This is a retrospective cohort study including children aged 0 months to 14 years, diagnosed with community acquired UTI (CA-UTI) at the Emergency Rooms (ERs) of the Department of Women's and Children's Health in Padova and at the Dell'Angelo Hospital in Mestre (Venice), between January 1st, 2016 and December 31st, 2018.

A CA-UTI episode was defined as all patient encounters with International Classification of Diseases, Ninth Revision, Clinical Modification diagnosis codes (ICD-9-CM codes: 590.x, 595.x, 599.0) OR free text corresponding to CA-UTI (Italian *infezione delle vie urinarie*, *pielonefrite*, *cistite*) identified in the electronic medical records of the hospital databases (Q-lik, Galileo and Aurora), AND a positive urine culture AND fever (body temperature ≥ 38 °C).

ER CA-UTI clinical assessments from the same patient occurring within 30 days from the first assessment were considered as follow-ups of the same episode.

A positive urine culture was defined as more than 10^4^ colony-forming units (CFU)/mL or as more than 10^5^ CFU/mL of an organism known to cause CA-UTI with urine collected by catheterization or clean-catch mid-stream /collection bag method, respectively.

All patients with a hospital admission in the previous 30 days were excluded from the study.

### Data collection

All clinical, demographic, and microbiological data were manually collected from electronic medical records, using a password protected REDCap 10.0.1-©2020 (Vanderbilt University) data collection form and stored in the secured server at the University of Padova. Privacy was guaranteed by assigning each patient a unique study number and no personal identifiable data were collected. Data collected included: date of birth, sex, diagnosis, clinical symptoms, antibiotic therapy in the previous 30 days, CA-UTI diagnosis in the previous 30 days, presence of exclusion criteria, date of urine collection, positivity for leukocyte esterase, positivity for nitrate, type of bacteria identified, resistance profile to different antibiotics.

Leukocyte esterase and nitrate detection was performed either with a dipstick by a healthcare worker or analyzed in the centres' laboratories. For both centres, bacteria isolates were identified by standard criteria, and antibiotic sensitivity was studied with the VITEK®2 system by Biomerieux (Marcy l'Etoile, France) using appropriate panels or a disc diffusion method following EUCAST [14] guidelines and breakpoints according to the centres' standard procedures.

### WISCA model

The tool was created based on pathogens isolated from patients with CA-UTIs. The most resistant culture was selected for patients with multiple positive urine cultures for the same isolate during one episode, and intermediate antibiotic sensitivity was considered resistant. Urine culture data with more than one pathogen were excluded since positively related to contamination.

We studied the antibiotic agents available on the centre/region formulary and for which automated sensitivity testing is routinely performed, and we grouped them in eight empirical treatments based on centre/national guidelines [15]: amikacin, co-amoxiclav, ampicillin-gentamicin, carbapenems (meropenem, imipenem, doripenem), III-gen. cephalosporins (cefixime, ceftibuten, ceftriaxone), fluoroquinolones (ciprofloxacin), piperacillin and tazobactam, co-trimoxazole. The only double combination treatment considered was ampicillin-gentamicin. In case of carbapenems and III-gen. cephalosporins, if a single molecule tested in the antibiogram was reported as resistant, then the empirical treatment was reported as resistant. In case of ampicillin-gentamicin, if one molecule tested in the antibiogram was reported as sensitive, then the empirical treatment was reported as sensitive.

### Statistical analysis

Continuous variables were described with the median and interquartile ranges (IQR) and categorical variables with percentages and absolute numbers. Differences in distributions of continuous variables were assessed using the Mann–Whitney U test, and χ2 or Fisher's exact test were used for categorical variables, as appropriate. The WISCA tool was implemented as a Bayesian logistic regression model to estimate the antibiotic regimens coverage of pathogens. Pathogens included in the WISCA are shown in Table 1. A hierarchical structure was specified, with varying intercepts for pathogens and empirical regimens, to provide stable and reliable coverage estimates, especially for the antibiotic regimens where a low number of pathogens tested for sensitivity [16, 17]. The following covariates were included: age group, sex and a binary variable that indicates if the subject had previous antibiotic treatment or renal/urological comorbidities (i.e., complex cases).

**Table 1:** Demographic characteristics of patient included with p value referred to the overall cohort stratified by complex cases (those who had previous antibiotic treatment or renal/urological comorbidities)

Complex case?	Centre A		Centre B		Overall		p value
	No	Yes	No	Yes	No	Yes	
	(n = 68)	(n = 23)	(n = 225)	(n = 69)	(n = 293)	(n = 92)	
Sex, N (%), male	32 (47.1%)	15 (65.2%)	93 (41.3%)	23 (33.3%)	125 (42.7%)	38 (41.3%)	0.972
Age, median [IQR], months	7.50 [13.3]	4.00 [11.5]	10.0 [25.0]	31.0 [63.5]	9.00 [21.0]	19.0 [55.5]	< 0.001
Age class, N (%)							
0–1 month	9 (13.2%)	6 (26.1%)	17 (7.6%)	3 (4.3%)	26 (8.9%)	9 (9.8%)	0.808
2–6 months	24 (35.3%)	9 (39.1%)	69 (30.7%)	9 (13.0%)	93 (31.7%)	18 (19.6%)	0.019
7–24 months	24 (35.3%)	4 (17.4%)	76 (33.8%)	18 (26.1%)	100 (34.1%)	22 (23.9%)	0.047
3–5 years	4 (5.9%)	3 (13.0%)	37 (16.4%)	17 (24.6%)	41 (14.0%)	20 (21.7%)	0.060
6–10 years	6 (8.8%)	1 (4.3%)	21 (9.3%)	17 (24.6%)	27 (9.2%)	18 (19.6%)	0.008
11–14 years	1 (1.5%)	0 (0%)	5 (2.2%)	5 (7.2%)	6 (2.0%)	5 (5.4%)	0.033
Discharge diagnosis, N (%)							
Urinary tract infection	50 (73.5%)	16 (69.6%)	177 (78.7%)	49 (71.0%)	227 (77.5%)	65 (70.7%)	0.251
Cystitis	8 (11.8%)	1 (4.3%)	40 (17.8%)	11 (15.9%)	48 (16.4%)	12 (13.0%)	0.347
Kidney infection	5 (7.4%)	5 (21.7%)	5 (2.2%)	8 (11.6%)	10 (3.4%)	13 (14.1%)	< 0.001
Other	5 (7.4%)	1 (4.3%)	3 (1.3%)	1 (1.4%)	8 (2.7%)	2 (2.2%)	0.762
Antibiotic prescription in the previous 30 days, N (%)							
Yes		12 (52.2%)		42 (60.9%)		54 (58.7%)	
Amikacin		0 (0%)		1 (1.4%)		1 (1.1%)	
Amoxicillin		0 (0%)		7 (10.1%)		7 (7.6%)	
Co-amoxiclav		2 (8.7%)		16 (23.2%)		18 (19.6%)	
Ampicillin		1 (4.3%)		0 (0%)		1 (1.1%)	
Azithromycin		0 (0%)		0 (0%)		0 (0%)	
Cefaclor		3 (13.0%)		1 (1.4%)		4 (4.3%)	
Cefixime		1 (4.3%)		8 (11.6%)		9 (9.8%)	
Cefpodoxime		0 (0%)		2 (2.9%)		2 (2.2%)	
Ceftriaxone		0 (0%)		1 (1.4%)		1 (1.1%)	
Ciprofloxacin		0 (0%)		4 (5.8%)		4 (4.3%)	
Co-trimoxazole		0 (0%)		0 (0%)		0 (0%)	
Fosfomycin		0 (0%)		1 (1.4%)		1 (1.1%)	
Missing		5 (41.7%)		1 (2.4%)		6 (8.8%)	
Previous treatment failure		0 (0%)		2 (2.9%)^a^		2 (2.2%)	
Presence of urinary tract malformations, N (%), Yes		15 (65.2%)		45 (65.2%)		60 (65.2%)	
Method for urine collection, N (%)							
Collection bag > 10^5^ CFU/ml	67 (98.5%)	23 (100%)	108 (48.0%)	23 (33.3%)	175 (59.7%)	46 (50.0%)	0.053
Catheterization > 10^4^ CFU/ml	0 (0%)	0 (0%)	30 (13.3%)	6 (8.7%)	30 (10.2%)	6 (6.5%)	0.277
Clean-catch mid-stream > 10^5^ CFU/ml	0 (0%)	0 (0%)	70 (31.1%)	37 (53.6%)	70 (23.9%)	37 (40.2%)	< 0.001
Missing	1 (1.5%)	0 (0%)	17 (7.2%)	3 (4.3%)	18 (5.9%)	3 (3.3%)	
Blood culture collection, N (%)							
Yes	22 (32.4%)	9 (39.1%)	65 (28.9%)	20 (29.0%)	87 (29.7%)	29 (31.5%)	0.667
Negative	21 (30.9%)	8 (34.8%)	28 (12.4%)	7 (10.1%)	49 (16.7%)	15 (16.3%)	0.256
Positive	1 (1.5%)	1 (4.3%)	1 (0.4%)	1 (1.4%)	2 (0.7%)	2 (2.2%)	
* Escherichia coli*	1 (1.5%)	1 (4.3%)	0 (0%)	0 (0%)	1 (0.3%)	1 (1.1%)	1
* Micrococcus luteus*	0 (0%)	0 (0%)	1 (0.4%)	0 (0%)	1 (0.3%)	0 (0%)	0.248
* Staphylococcus epidermidis*	0 (0%)	0 (0%)	0 (0%)	1 (1.4%)	0 (0%)	1 (1.1%)	0.248
Urine culture bacteria, N (%)							
* Citrobacter koseri*	0 (0%)	0 (0%)	2 (0.9%)	0 (0%)	2 (0.7%)	0 (0%)	0.425
* Enterobacter cloacae*	0 (0%)	0 (0%)	3 (1.3%)	0 (0%)	3 (1.0%)	0 (0%)	0.328
* Enterococcus faecalis*	1 (1.5%)	0 (0%)	2 (0.9%)	0 (0%)	3 (1.0%)	0 (0%)	0.328
* Escherichia coli*	58 (85.3%)	21 (91.3%)	196 (87.1%)	55 (79.7%)	254 (86.7%)	76 (82.6%)	0.277
* Klebsiella oxytoca*	2 (2.9%)	0 (0%)	0 (0%)	2 (2.9%)	2 (0.7%)	2 (2.2%)	0.222
* Klebsiella pneumoniae*	1 (1.5%)	0 (0%)	1 (0.4%)	3 (4.3%)	2 (0.7%)	3 (3.3%)	0.058
* Proteus mirabilis*	5 (7.4%)	0 (0%)	19 (8.4%)	5 (7.2%)	24 (8.2%)	5 (5.4%)	0.373
* Proteus vulgaris*	0 (0%)	0 (0%)	1 (0.4%)	0 (0%)	1 (0.3%)	0 (0%)	0.573
* Pseudomonas aeruginosa*	0 (0%)	2 (8.7%)	0 (0%)	3 (4.3%)	0 (0%)	5 (5.4%)	< 0.001
* Staphylococcus aureus*	1 (1.5%)	0 (0%)	1 (0.4%)	1 (1.4%)	2 (0.7%)	1 (1.1%)	0.705

^a^Co-amoxiclav + fosfomycin

Differences in coverage between centers were evaluated using Bayesian Leave-One-Out cross-validation and computing the differences between Expected Log-Predictive Densities (ELPDs) of the models with and without center effect [18]. Differences were considered statistically significant if the 95% Confidence Interval of the ELPDs difference did not include the zero value [19].

The Hamiltonian Monte Carlo algorithm was employed to sample from the posterior distribution of the parameters using Stan software for Bayesian inference [20]. The posterior distributions of the parameters and the different coverages were summarized using the median and the 95% Highest Density Intervals (HDIs). Wider HDIs reflect uncertainty in the coverage. Differences between age groups, sex, and complex cases were expressed as Odds Ratios (ORs) with relative 95% HDIs. More technical details on the specification and the model's implementation can be found in the Additional file 1.

The statistical analysis was implemented using R software for statistical computing (version 4.0.0). [21] The model was fitted with brms R package (version 2.12) [22] and model comparison was performed using loo R package (version 2.2.0). [23]

## Results

**Fig. 1:**
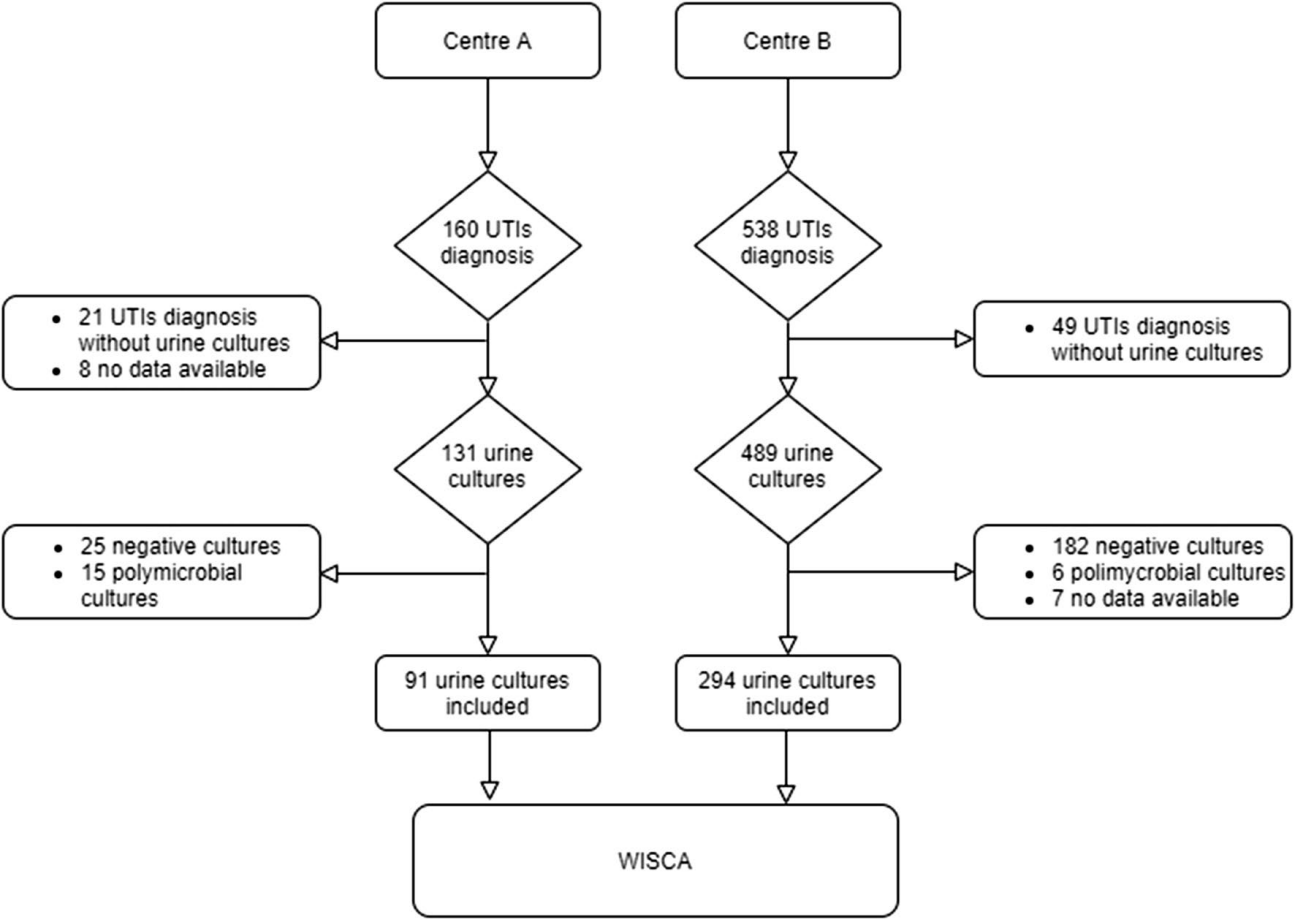
Flowchart of case selection according to Strengthening the Reporting of Observational Studies in Epidemiology (STROBE) guidelines

Each centers was randomly named with a capital letter (Centre A and B) to maintain anonymity. Included episodes are summarized in Fig. 1.

### Population characteristics

Demographic characteristics are summarised in Table 1 and stratified according to previous antibiotic treatment or renal/urological comorbidities and centre. Overall, children in the complex case group were older (19.0 (IQR: 55.5) versus 9.0 (IQR:21) months of age in the complex versus non-complex group, respectively; P < 0.001). Urine samples were mainly collected with a collection bag or a clean-catch mid-stream method, in the latter case with significant differences in the complex versus non-complex group (40.2% vs. 23.9% respectively; P < 0.001*). E. coli* and *Proteus* spp. were the most prevalent pathogens (86.7% vs. 82.6% for *E. coli* and 8.2% vs. 5.4% for *Proteus* spp. for non-complex and complex cases, respectively). *Pseudomonas aeruginosa* was found only in samples collected in the complex case group (5.4%).

### WISCA results

The algorithm used to sample from the parameters posterior distributions achieved an optimal, with R ^ index values always near 1 and a good mixing for all the chains. More details on the algorithm's convergence can be found in Table S1 and Figure S1 (Additional file 1). The posterior distributions of the model's parameters are shown in Figure S2 (Additional file 1).

There were no significant differences found in the models' predictive performances with and without centre effect, resulting in an Expected Log-Predictive Densities difference of -0.87 (95% CI, -4.47; 2.74). Therefore, we reported the coverage estimates pooling data from the two centres.

**Fig. 2:**
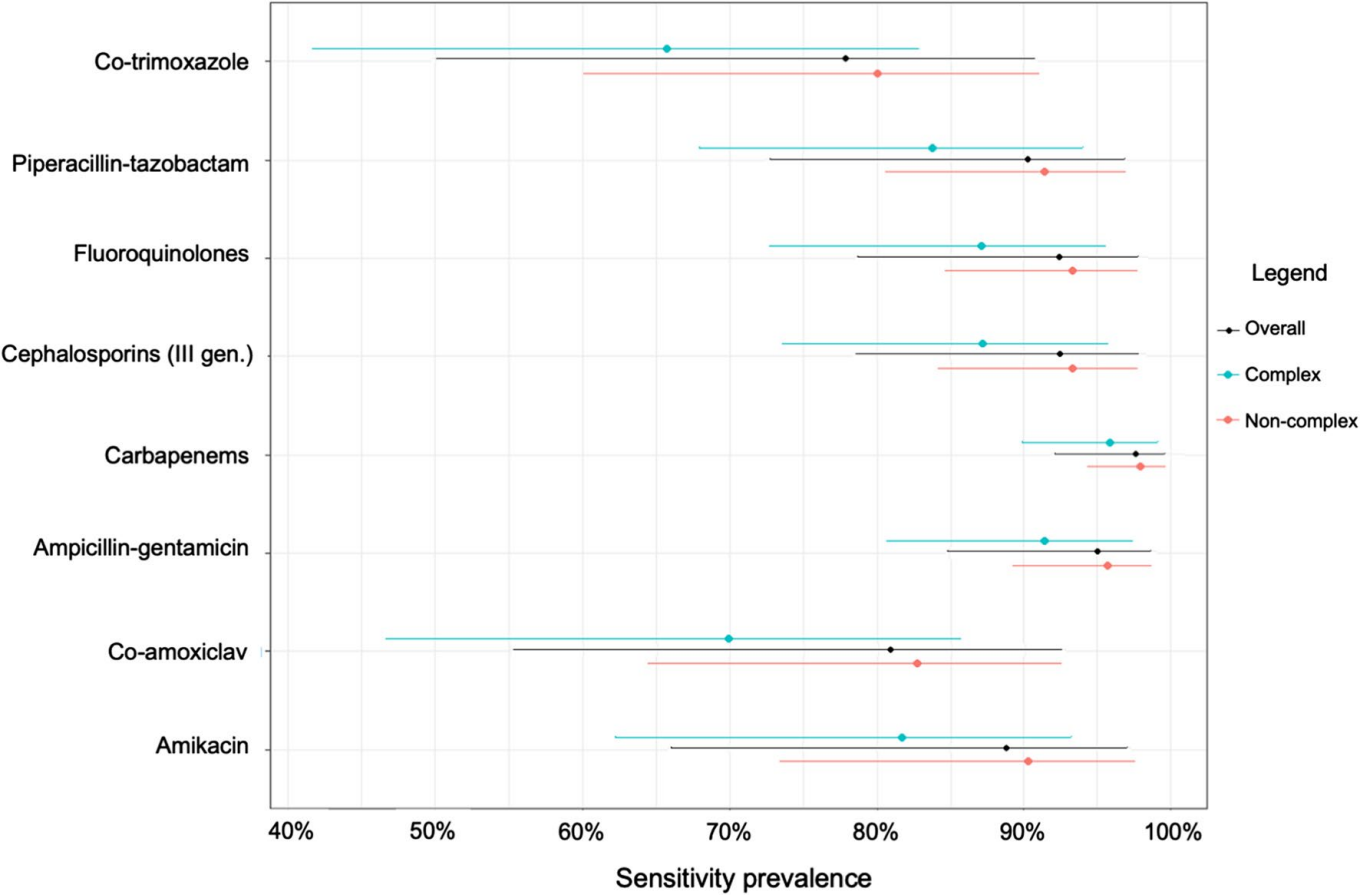
WISCA estimated coverage for all the evaluated antibiotic regimes. Dots represent the median of the posterior distribution and line the associated 95% Highest Density Intervals

In Fig. 2 and Table S2 (Additional file 1), the WISCA estimated coverage for all the treatment regimens is shown along with their relative 95% Highest Density Intervals (HDI).

Estimates range from 77.8% (Co-trimoxazole) to 97.6% (Carbapenems). Large 95% HDI reflects the high uncertainty surrounding the coverage for those antibiotic regimes with a low number of pathogens identified or tested, i.e., Co-trimoxazole (95% HDI, 50.1%–90.7%).

Table S3 (Additional file 1) also shows WISCA estimated coverage for each antibiotic regimen stratified by complex and non-complex group and by sex, respectively. Complex cases showed lower coverage for all the treatment regimens, with significantly lower odds of being covered by treatments than non-complex cases (Odds Ratio (OR) 0.49 [95% HDI, 0.38 – 0.65]). Males had significantly lower odds of being covered by an antibiotic regimen than females, with an OR of 0.73 (95% HDIs, 0.56–0.96).

**Fig. 3:**
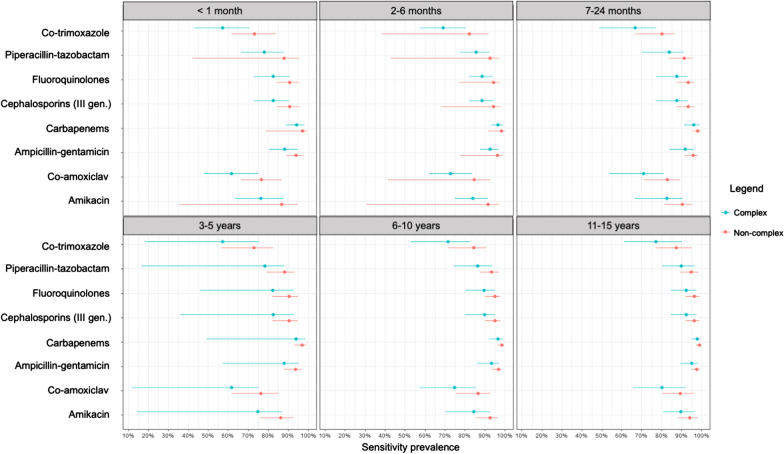
WISCA estimated coverage for all the evaluated antibiotic regimes stratified by age group and non-complex versus complex cases. Dots represent the median of the posterior distribution and the line the associated 95% Highest Density Intervals

Figure 3 and Table S3 (Additional file 1) show the estimated coverage for age groups.

The lowest estimated coverage was observed for neonates and children aged 3–5 years. The latter age class had lower odds of being covered by an antibiotic treatment than children aged 2–6 months (OR: 1.84 [95% HDI, 1.18–2.69]), 6–10 years (OR:1.89 [95% HDI, 1.26–3.21]) and 11–15 years (OR: 2.32 [95% HDI, 1.18–7.08]), especially the non-complex cohort (Table S4—Additional file 1).

## Discussion

To our knowledge, this is the first study led in Italy developing a WISCA aiming to guide the choice of the most suitable empiric antibiotic treatment for CA-UTIs in paediatric outpatients. Data from two centres were successfully pooled to predict the treatment coverage and were further stratified according to the presence of previous antibiotic treatment or renal/urological comorbidities, age groups, and sex. This allowed us to maximize the available data and enable us to partly overcome the limitations of the reduced sample numerosity in the different strata.

Today there are still analytical challenges in developing WISCAs for the paediatric population represented mainly by the paucity of data that can be overcome using Bayesian methods. We proposed a WISCA tool that expands the framework of the classic hospital combined antibiograms, providing weighted coverage estimates based on the frequency of the pathogens identified, and of the WISCA algorithms recommended in previous studies [10–12, 25]. Classically combined antibiograms usually have only one level of stratification, predominately based on the hospital ward, and then, if the sample is big enough, the second stratification is based on broad age groups (i.e., usually children vs. adult vs. elderly—see Fig. S3 in the Additional file 1). Rarely the combined antibiogram is stratified for sex and comorbilities/previous antibiotic treatments, as in our case [7, 25]. Moreover, the combined antibiogram does not distinguish among asymptomatic bacteriuria, colonization and infection [26].

Our approach presents two main new features from a methodological point-of-view. First, we specified a Bayesian hierarchical logistic regression with random effects structures on the pathogens and the treatment regimens. The hierarchical structure choice was motivated by the need to provide reliable coverage estimates that can aid the clinician in choosing the optimal antibiotic regimen. Second, we included covariates in the model that allow profiling coverage estimates in terms of children's characteristics, such as age, sex, and previous antibiotic treatment or renal/urological comorbidities.

We demonstrated that children with previous antibiotic treatment or renal/urological comorbidities had significantly lower odds of being covered by an antibiotic treatment compared to non-complex cases. Moreover, different variations in the coverage were observed, stratifying the WISCAs according to age groups. Children aged 3–5 years old had lower odds of being covered by the treatments analyzed than other age groups, with the exception of neonates.

In line with the literature, *E. coli* and *P. mirabilis* were the most frequently found pathogens. In a study analyzing children's urine culture data retrospectively from 2007 to 2014 in a paediatric hospital in the north of Italy [27], *E. coli* was the most prevalent pathogen from ER Gram-negative samples (75.0%) and *Pseudomonas* spp. accounted for just 2.2% of positivity. In the same study, data from different departments were pooled to investigate the resistance pattern to oral antibiotics. The authors reported that *E. coli* resistance to co-amoxiclav, cefuroxime, and ciprofloxacin increased significantly from 2007–2010 to 2011–2014 and that resistance to beta-lactams was most frequent in males older than one year, with more than one CA-UTI episode and followed by hospital Departments dealing with urinary tract malformations.

Despite being very informative, data from inpatients and outpatients should not be pooled because of possible hospital-acquired infections mainly constituted by MDRO, causing an overestimation of resistance pattern. A study conducted in 2009 reporting *E. coli*, *S. aureus* and *S. pneumoniae* sensitivity data from inpatients and outpatients in a USA hospital demonstrated significant differences in resistance patterns in the two settings, especially for beta-lactams agents. [7], For this reason, WISCA tool for hospital acquired urinary tract infection (i.e., catheter related infection) need to be developed with separate estimates.

In Italy, beta-lactams represent the preferred therapy for children with CA-UTIs, with co-amoxiclav being recommended as first-line and III-gen. cephalosporins as second-line in selected cases [15]. In our study the estimated coverage to amoxicillin varied on the basis of strata: in the overall WISCA the coverage was 80.8% [55.2%–92.4%], increasing for non-complex cases to 82.7% [64.4%–92.6%] and decreasing for complex ones to 69.9% [46.7%–85.7%]. Our findings are in line with a study analyzing urine culture coverage data from 2016 to 2017 from an ER in the USA and stratifying the cohort based on previous antibiotic treatment and/or the presence of renal or urological comorbidities/malformations and/or prior hospital admission: co-amoxiclav coverage varied significantly from 86.2% to 72.1% in the healthy and complex cohort respectively [25].

Previous antibiotic therapy represents a relevant factor in defining the resistance pattern in CA-UTIs and it has been demonstrated that the magnitude of this association decreases with time following exposure [28]. Amoxicillin exposure within 30 days before the onset of a CA-UTI was associated with an almost four times higher probability of resistance to co-amoxiclav in previously healthy children. This finding is especially important when considering data from different age groups. Co-amoxiclav coverage was the lowest in children aged 3–5 years in the complex cohort, followed by neonates and children aged 6–24 months. In Italy, the prevalence of antibiotic prescriptions is the highest in pre-schoolers. More than 30% of children receive 2 or more prescriptions a year, increasing the chances of developing antibiotic resistance [29]. The lower coverage in neonates may be associated with the vertical transmission of the mother's pathogen resistance during labour, as found previously [30–32].

The presence of renal/urological comorbidities increases the risk of antibiotic resistance related to antibiotic prophylaxis, the presence of a urinary catheter, and a higher risk of MDRO colonization. [27]

### Strengths and limitations

The strength of our study is that we pooled data from two different centres to create a stratified WISCA specific for paediatric patients. First, we successfully pooled urine culture data from the emergency rooms of two hospitals in the North of Italy in order to develop a WISCA specific for CA-UTI in paediatric patients stratifying data according to patients’ previously undertaking antibiotic treatment, the presence of renal/urological co-morbidities, as well as age class. Second, we combined clinical data with microbiological data allowing us to produce more precise coverage estimates, which confirmed first-line empiric treatment validity recommended by CA-UTIs guidelines. Third, it is possible to extend WISCA to hospitals and primary care ambulatories located within the two Centres, in order to support clinicians in decision-making around the most appropriate empirical approach, for example through the development of clinical pathways. Fourth, our methodology can be applied by other centres, with the final aim of improving the prescribing behaviour in clinical practice.

A limitation of this study is related to its retrospective nature. Data on six antibiograms were not available; however, we have no reason to believe that those unavailable data would have differed significantly from the data included in the cohort. It may also be argued that we did not consider antibiotic prescriptions up to sixty days before the ER visits, which might have caused a possible overestimation of resistance in the non-complex cohort. On the other hand, it is not a common clinical practice to assess antibiotics prescribed more than one month before the clinical assessment; thus our criteria are more in line with the setting considered. Furthermore, the WISCA model does not take into account the safety of the regimen in regards to possible adverse events such as beta-lactams allergy or ototoxicity associated with gentamicin treatment. Whenever prescribing an empiric antibiotic regimen, the individual characteristics of the patient must be taken into account. Finally, we did not include inpatient data where MDRO colonization increases the challenge in defining the most appropriate treatment.

## Conclusions

The developed WISCAs provide highly informative estimates on coverage patterns overcoming the limitation of combination antibiograms and expanding the framework of previous Bayesian WISCA algorithm. Moreover, it represents a valid tool in monitoring antibiotics resistance data, and it may help in re-evaluating the first-line treatment for local guidelines or clinical pathways.

## Supplementary Information

**Additional file 1.** Supplementary Figures and Tables.

## Data Availability

The dataset supporting the conclusions of this article are available upon a reasonable request to the corresponding author.

## Abbreviations

CFU: Colony Forming Units
CI: Confidence intervals
ECDC: European Centre for Disease Prevention and Control
ELPD: Expected Log-Predictive Densities
ER: Emergency Room
HDI: Highest Density Interval
ICD-9-CM: International Classification of Diseases, Ninth Revision, Clinical Modification
MDRO: Multi drug-resistant organism
OR: Odds Ratio
CA-UTI: Community acquired urinary tract infection
WISCA: Weighted Incidence Syndromic Combination Antibiogram
