# Supplementary material for: Development of a Weighted-Incidence Syndromic Combination Antibiogram (WISCA) to guide the choice of the empiric antibiotic treatment for urinary tract infection in paediatric patients: a Bayesian approach
Source: Antimicrob Resist Infect Control. 2021 May 1;10:74. doi: 10.1186/s13756-021-00939-2 (PMC8088309; doi:10.1186/s13756-021-00939-2)

***Additional file1***

**WISCA model**

Adapted from Bielicki et al, the WISCA was developed as a decision tree (Figure 1a), with the first node (circle) representing the clinical decision to initiate empiric treatment, the second node representing all the possible bacteria causing the infection and the third node representing the possible susceptibility profile of the bacteria (dichotomous: susceptible/resistant).

Figure 1a. WISCA model (adapted from Bielicki et al.)


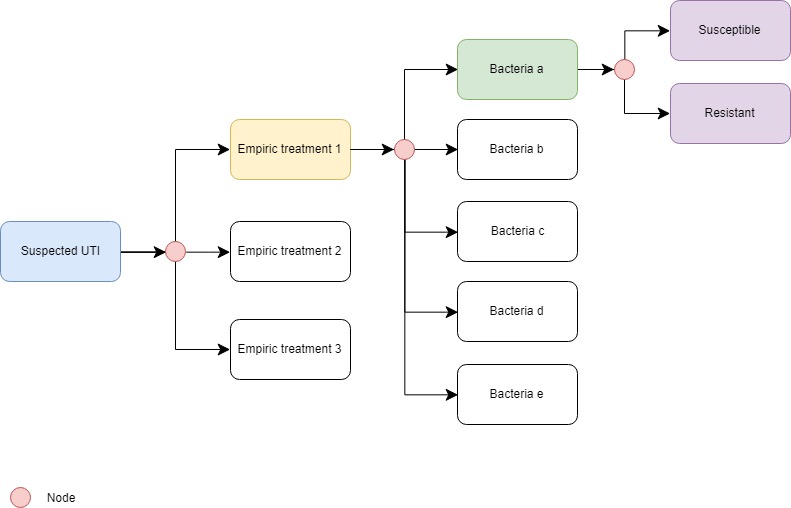


**Bayesian WISCA hierarchical model**

A Bayesian hierarchical logistic regression model was used to develop the WISCA predictive tool. In detail, we imposed a random-effects structure both on the pathogens and the treatment regimens. Considering $i = 1, \ldots, N$ observations in the data, $k = 1, \ldots, K$ pathogens, and $j = 1, \ldots, J$ treatment regimens, the model was specified as follows:

$$P\left( coverage_{i}=1 \right)=logit^{-1}\left( \alpha_{0}+\alpha_{k\left[ i \right]}+\alpha_{j\left[ i \right]}+\beta X_{i} \right)$$

where:

- $\alpha_{0}$ represents the overall intercept of the model
- $\alpha_{k}$ represents the random effects structure imposed on the pathogens, i.e. the random variation of the pathogens intercepts from the overall intercept
- $\alpha_{j}$ represents the random effects structure imposed on the treatment regimens, i.e. the random variation of the treatment regimens intercepts from the overall intercept
- $\beta$ represents the vector of the fixed effects coefficients, which includes the coefficients related to the sex, the complex cases and the age groups
- $X$ represents the fixed effects design matrix
- the subscripts $k\left[ i \right]$ and $j\left[ i \right]$ represent the *k-th* pathogen for the *i-th* observation, and the *j-th* regimen for the *i-th* observation, respectively

We imposed the following prior distributions on the parameters of the model:

$$\alpha_{0}\sim T-student\left( 3,0,10 \right)$$

$$\alpha_{k}\sim N\left( 0,\sigma_{pathogen} \right)$$

$$\alpha_{j}\sim N\left( 0,\sigma_{regimen} \right)$$

$$\beta\sim T-student\left( 3,0,1 \right)$$

$$\sigma_{pathogen}\sim{T-student}^{+}\left( 3,0,1 \right)$$

$$\sigma_{regimen}\sim{T-student}^{+}\left( 3,0,1 \right)$$

The choice to impose a hierarchical structure on pathogens and treatment regimens was motivated by the advantages that such a model’s specification provides in terms of inference on the treatment regimen’s coverage. More specifically, the intercepts associated with pathogens and regimens are conceptualized as draws from a normal distribution with mean equal to the overall intercept and standard deviations. The prior distributions on the standard deviations control the variability of the intercepts around the common mean. We chose to specify “weakly informative” prior distributions on the standard deviation parameters. Such class of priors has nice regularizing properties, i.e. less probability of occurrence is given to values that are too extreme to be observed in practice. [1,2] Thus, the weakly informative priors prevent the estimate’s variability to be too large, such that the inference on the parameters is stable and reliable. [3] As a consequence, the coverage estimates will be less variable, and inference will be possible even for those combinations of pathogens and regimes for which the data are scarce, as occurred in our study. This may provide many advantages from a practical point-of-view since a stable regimen’s coverage estimate for each pathogen can provide more meaningful information to clinicians when they must choose the optimal antibiotic regimen. Weakly informative priors distributions were also imposed on the fixed effects parameters of the model, i.e. sex, complex cases, and groups of age. [4] The inclusion of the covariates in the model allows for stratification of the regimen coverage, providing risk profiles for children according to their characteristics.

Differences in coverage between centers were evaluated using Bayesian Leave-One-Out cross-validation and computing the differences between Expected Log-Predictive Densities (ELPDs) of the models with and without center effect (model with Centre effect ELPD minus model without Centre effect ELPD). [26] Differences were considered statistically significant if the 95% Confidence Interval of the ELPDs difference did not include the zero. [5]

The Hamiltonian Monte Carlo algorithm was employed to sample from the posterior distribution of the parameters using Stan software for Bayesian inference. [6] The algorithm was implemented with 4 chains and 3000 iterations were run on each chain, discarding the first 1000 as a warm-up. The convergence of the algorithm was checked using traceplots and R ̂ index. [7] The posterior distributions of the parameters and the different coverages were summarized using the median and the 95% Highest Density Intervals (HDIs). The choice of the median and the 95% HDIs to summarize the posterior distributions is motivated by the fact that they are robust to potential skewness in the distributions. Differences between age groups, sex, and complex cases were expressed as Odds Ratios (ORs) with relative 95% HDIs.

**Comparison of the WISCA tools**

The first WISCA tool proposed by Herbert et al. [8] aimed at providing antibiotic regimen coverage for several pathogens in subjects with ABI and UTI. Bielicki et al. [9] and Tandogdu et al. [10] expanded the tool using a Bayesian approach, which allowed the estimation of regimen coverage uncertainty and overcame the analytical challenges presented by the combination of regimens and pathogens for which there is a paucity of data. Bielicki and colleagues modeled regimen coverages with a binomial distribution and pathogens data with a multinomial distribution. They imposed non-informative prior distributions on the parameters of the model, giving more weights on the observed data to final inference on the estimated coverage. Tandogdu and colleagues adopted a similar modeling framework and they derived informative prior distribution on the parameters of the model using historical data. Our approach aims to expand the modeling framework of the WISCA tool to provide a more flexible description of the phenomenon. As in Bielicki et al. and Tandogdu et al., we developed the tool in a Bayesian framework. We built a hierarchical multilevel logistic regression model with random effects structure on pathogens and regimens to control the variability of the estimates and, thus, to provide more reliable information on the antibiotic regimen coverage for each pathogen. Moreover, the inclusion of covariates in the model allows for a detailed estimation of coverage in children with different characteristics, an aspect that was not accounted for in the previous study.

**References**

1. Gelman, A.; Simpson, D.; Betancourt, M. The Prior Can Often Only Be Understood in the Context of the Likelihood. *Entropy* **2017**, *19*, 555, doi:10.3390/e19100555.

2. Gabry, J.; Simpson, D.; Vehtari, A.; Betancourt, M.; Gelman, A. Visualization in Bayesian workflow. *Journal of the Royal Statistical Society: Series A (Statistics in Society)* **2019**, *182*, 389–402, doi:10.1111/rssa.12378.

3. Gelman, A.; Carlin, J.B.; Stern, H.S.; Dunson, D.B.; Vehtari, A.; Rubin, D.B. *Bayesian Data Analysis*; Texts in Statistical Sciences; Third Edition.; Chapman and Hall/CRC, 2013;

4. Gelman, A.; Jakulin, A.; Pittau, M.G.; Su, Y.-S. A weakly informative default prior distribution for logistic and other regression models. *Ann. Appl. Stat.* **2008**, *2*, 1360–1383, doi:10.1214/08-AOAS191.

5. Vehtari, A.; Gelman, A.; Gabry, J. Practical Bayesian model evaluation using leave-one-out cross-validation and WAIC. *Stat Comput* **2017**, *27*, 1413–1432, doi:10.1007/s11222-016-9696-4.

6. Carpenter, B.; Gelman, A.; Hoffman, M.D.; Lee, D.; Goodrich, B.; Betancourt, M.; Brubaker, M.; Guo, J.; Li, P.; Riddell, A. Stan: A Probabilistic Programming Language. *Journal of Statistical Software* **2017**, *76*, 1–32, doi:10.18637/jss.v076.i01.

7. Vehtari, A.; Gelman, A.; Simpson, D.; Carpenter, B.; Bürkner, P.-C. Rank-normalization, folding, and localization: An improved $\widehat{R}$ for assessing convergence of MCMC. *arXiv:1903.08008 [stat]* **2020**.

8. Hebert, C.; Ridgway, J.; Vekhter, B.; Brown, E.C.; Weber, S.G.; Robicsek, A. Demonstration of the weighted-incidence syndromic combination antibiogram: an empiric prescribing decision aid. *Infect Control Hosp Epidemiol* **2012**, *33*, 381–388, doi:10.1086/664768.

9. Bielicki, J.A.;; Sharland, M.;; Johnson, A.P.; Henderson, K.L.; Cromwell, D.A.; on behalf of the ARPEC project.; Selecting appropriate empirical antibiotic regimens for paediatric bloodstream infections: application of a Bayesian decision model to local and pooled antimicrobial resistance surveillance data. *J Antimicrob Chemother* **2016**, *71*, 794–802, doi:10.1093/jac/dkv397.

10. Tandogdu, Z.; Kakariadis, E.T.A.; Naber, K.; Wagenlehner, F.; Bjerklund Johansen, T.E. Appropriate empiric antibiotic choices in health care associated urinary tract infections in urology departments in Europe from 2006 to 2015: A Bayesian analytical approach applied in a surveillance study. *PLoS ONE* **2019**, *14*, e0214710, doi:10.1371/journal.pone.0214710.

**Tables and Figures**

Table 1s. R ̂ index values of the WISCA model’s parameters. The index is a convergence diagnostic of the Monte Carlo Markov Chain algorithm. It compares the between and within chains estimates of the parameters of the model. If the chains have mixed well, the values are always closer to 1. The parameter names that start with *b* identify the fixed effects coefficients, whereas standard deviation and varying intercepts parameter names start with *sd* and *r*, respectively.

Table 2s. WISCA estimated coverage (expressed as percentages) for all the evaluated treatment regimen presented as median of the posterior distributions and the associated 95% HDIs for the overall cohort and stratified by complex cases (those who had previous antibiotic treatment or renal/urological comorbidities).

Table 3s. WISCA estimated coverage (expressed as percentages) for all the evaluated treatment regimens presented as median of the posterior distributions and the associated 95% HDIs stratified by complex cases (those who had previous antibiotic treatment or renal/urological comorbidities) in groups of age.

Table 4s. Odds Ratio of coverage by groups of age with the associated 95%HDI assessing the probability with 3-5 years as reference category.

Figure 1s. Monte Carlo Markov Chains (MCMC) trace plots of the WISCA model’s parameters.

Figure 2s. Density plots of the posterior distributions of the parameters of the model. The parameter names that start with *b* identify the fixed effects coefficients, whereas standard deviation and varying intercepts parameter names start with *sd* and *r*, respectively.

Figure 3s. Example of a E. coli cumulative antibiogram of urine samples stratified by ward (General Pediatric) and age class (1-14 years) available at Centre B that describes susceptibility rates (expressed as percentage) for different antibiotics tested including data from January 2018 to June 2018.

*Table 1s. R ̂ index values of the WISCA model’s parameters. The index is a convergence diagnostic of the Monte Carlo Markov Chain algorithm. It compares the between and within chains estimates of the parameters of the model. If the chains have mixed well, the values are always closer to 1. The parameter names that start with b identify the fixed effects coefficients, whereas standard deviation and varying intercepts parameter names start with sd and r, respectively.*

| ***Parameters*** | ***R ̂ index values*** |
| --- | --- |
| *b_Intercept* | 0.9999954 |
| *b_age_cat2_6_m* | 0.9999352 |
| *b_age_cat6_m_2_y* | 1.0003449 |
| *b_age_cat3_5_y* | 1.0007542 |
| *b_age_cat6_10_y* | 0.9997941 |
| *b_age_cat11_15_y* | 0.9999770 |
| *b_sexmale* | 1.0003942 |
| *b_complex_caseYes* | 1.0000205 |
| *sd_antibiogram__Intercept* | 1.0004712 |
| *sd_organism__Intercept* | 1.0001549 |
| *r_antibiogram[amikacina,Intercept]* | 1.0003813 |
| *r_antibiogram[amox_clav,Intercept]* | 1.0004211 |
| *r_antibiogram[amp_gent,Intercept]* | 1.0002207 |
| *r_antibiogram[carbapenem,Intercept]* | 1.0000569 |
| *r_antibiogram[cefalo,Intercept]* | 1.0005213 |
| *r_antibiogram[fluoro,Intercept]* | 1.0002866 |
| *r_antibiogram[pip_taz,Intercept]* | 1.0001414 |
| *r_antibiogram[trim_sulfa,Intercept]* | 1.0002674 |
| *r_organism[Citrobacter.koseri,Intercept]* | 0.9997649 |
| *r_organism[Enterobacter.cloacae,Intercept]* | 0.9996282 |
| *r_organism[Enterococcus.faecalis,Intercept]* | 0.9998856 |
| *r_organism[Escherichia.coli,Intercept]* | 0.9998327 |
| *r_organism[Klebsiella.spp,Intercept]* | 0.9997876 |
| *r_organism[Proteus.spp,Intercept]* | 0.9997206 |
| *r_organism[Pseudomonas.aeruginosa,Intercept]* | 0.9999786 |
| *r_organism[Staphylococcus.aureus,Intercept]* | 0.9997881 |

Table2s. WISCA estimated coverage (expressed as percentages) for all the evaluated treatment regimen presented as median of the posterior distributions and the associated 95% HDIs for the overall cohort and stratified by complex cases (those who had previous antibiotic treatment or renal/urological comorbidities) and sex, respectively.

| ***Antibiotic*** | ***Overall*** | ***Complex cases*** | | ***Sex*** | |
| --- | --- | --- | --- | --- | --- |
|  |  | ***No*** | ***Yes*** | ***Female*** | ***Male*** |
| *Amikacin* | 88.9 [66.1 - 97.1] | 90.3 [73.5 - 97.5] | 81.7 [62.2 - 93.2] | ***90.5 [76.3 - 94.8]*** | ***86.6 [41.2 - 92.8]*** |
| *Co-amoxiclav* | 80.8 [55.2 - 92.4] | 82.7 [64.4 - 92.6] | 69.9 [46.7 - 85.7] | ***82.8 [63.7 - 89.7]*** | ***77.7 [43.9 - 86.2]*** |
| *Ampicillin-gentamicin* | 95.0 [84.9 - 98.7] | 95.6 [89.3 - 98.7] | 91.4 [80.7 - 97.4] | ***95.6 [88.4 - 97.8]*** | ***94.1 [78.0 - 96.9]*** |
| *Carbapenems* | 97.6 [92.1 - 99.6] | 97.9 [94.4 - 99.5] | 95.9 [89.9 - 99.1] | ***97.9 [94.2 - 99.1]*** | ***97.1 [88.0 - 98.8]*** |
| *Cephalosporins (III gen.)* | 92.4 [78.6 - 97.8] | 93.3 [84.2 - 97.8] | 87.2 [73.6 - 95.7] | ***93.3 [83.1 - 96.4]*** | ***91.1 [70.3 - 95.1]*** |
| *Fluoroquinolones* | 92.4 [78.7 - 97.8] | 93.3 [84.6 - 97.8] | 87.1 [72.7 - 95.6] | ***93.3 [83.0 - 96.4]*** | ***91.0 [71.8 - 95.0]*** |
| *Piperacillin- tazobactam* | 90.2 [72.8 - 96.9] | 91.4 [80.6 - 96.9] | 83.8 [68.0 - 94.1] | ***91.5 [79.1 - 95.3]*** | ***88.3 [54.6 - 93.5]*** |
| *Co-trimoxazole* | 77.8 [50.1 - 90.7] | 80.0 [60.1 - 91.1] | 65.8 [41.7 - 82.8] | ***80.1 [58.7 - 87.8]*** | ***74.5 [39.4 - 83.8]*** |

Table 3s. WISCA estimated coverage (expressed as percentages) for all the evaluated treatment regimens presented as median of the posterior distributions and the associated 95% HDIs stratified by complex cases (those who had previous antibiotic treatment or renal/urological comorbidities) in groups of age. (continued in the next page)

| ***Antibiotic*** | ***Age groups*** | | | | | |
| --- | --- | --- | --- | --- | --- | --- |
|  | ***<1 month*** | | ***2-6 months*** | | ***7-24 months*** | |
|  | ***Non-complex*** | ***Complex*** | ***Non-complex*** | ***Complex*** | ***Non-complex*** | ***Complex*** |
| *Amikacin* | 0.87 [0.36 - 0.95] | 0.76 [0.64 - 0.87] | 0.92 [0.31 - 0.97] | 0.84 [0.75 - 0.91] | 0.90 [0.82 - 0.95] | 0.82 [0.67 - 0.90] |
| *Co-amoxiclav* | 0.77 [0.66 - 0.87] | 0.62 [0.48 - 0.75] | 0.85 [0.41 - 0.93] | 0.73 [0.62 - 0.83] | 0.83 [0.71 - 0.89] | 0.71 [0.54 - 0.81] |
| *Ampicillin-gentamicin* | 0.94 [0.89 - 0.97] | 0.88 [0.81 - 0.94] | 0.96 [0.78 - 0.99] | 0.93 [0.88 - 0.97] | 0.96 [0.91 - 0.98] | 0.92 [0.84 - 0.96] |
| *Carbapenems* | 0.97 [0.79 – 1.00] | 0.94 [0.89 - 0.98] | 0.98 [0.92 – 1.00] | 0.96 [0.93 - 0.99] | 0.98 [0.95 - 0.99] | 0.96 [0.91 - 0.99] |
| *Cephalosporins (III gen.)* | 0.91 [0.85 - 0.96] | 0.83 [0.73 - 0.90] | 0.94 [0.68 - 0.98] | 0.89 [0.82 - 0.94] | 0.93 [0.87 - 0.96] | 0.88 [0.77 - 0.93] |
| *Fluoroquinolones* | 0.91 [0.84 - 0.96] | 0.83 [0.73 - 0.91] | 0.94 [0.77 - 0.98] | 0.89 [0.82 - 0.94] | 0.93 [0.87 - 0.96] | 0.88 [0.77 - 0.93] |
| *Piperacillin- tazobactam* | 0.88 [0.42 - 0.96] | 0.78 [0.66 - 0.88] | 0.93 [0.43 - 0.97] | 0.86 [0.78 - 0.92] | 0.91 [0.84 - 0.95] | 0.84 [0.70 - 0.91] |
| *Co-trimoxazole* | 0.73 [0.62 - 0.84] | 0.57 [0.43 - 0.71] | 0.82 [0.39 - 0.92] | 0.69 [0.57 - 0.81] | 0.80 [0.67 - 0.86] | 0.67 [0.49 - 0.77] |

| ***Antibiotic*** | ***Age groups*** | | | | | |
| --- | --- | --- | --- | --- | --- | --- |
|  | ***3-5 years*** | | ***6-10 years*** | | ***11-15 years*** | |
|  | ***Non-complex*** | ***Complex*** | ***Non-complex*** | ***Complex*** | ***Non-complex*** | ***Complex*** |
| *Amikacin* | 0.86 [0.76 - 0.93] | 0.75 [0.14 - 0.87] | 0.93 [0.85 - 0.97] | 0.84 [0.70 - 0.93] | 0.94 [0.88 - 0.98] | 0.89 [0.81 - 0.97] |
| *Co-amoxiclav* | 0.76 [0.62 - 0.85] | 0.62 [0.12 - 0.75] | 0.87 [0.76 - 0.93] | 0.75 [0.57 - 0.86] | 0.89 [0.80 - 0.96] | 0.80 [0.66 - 0.92] |
| *Ampicillin-gentamicin* | 0.94 [0.88 - 0.97] | 0.88 [0.57 - 0.95] | 0.97 [0.93 - 0.99] | 0.93 [0.86 - 0.97] | 0.97 [0.95 - 0.99] | 0.95 [0.90 - 0.98] |
| *Carbapenems* | 0.97 [0.93 - 0.99] | 0.94 [0.49 - 0.98] | 0.98 [0.96 – 1.00] | 0.97 [0.92 - 0.99] | 0.99 [0.97 – 1.00] | 0.98 [0.95 - 0.99] |
| *Cephalosporins (III gen.)* | 0.90 [0.82 - 0.95] | 0.83 [0.36 - 0.93] | 0.95 [0.90 - 0.98] | 0.90 [0.80 - 0.95] | 0.96 [0.92 - 0.99] | 0.92 [0.85 - 0.97] |
| *Fluoroquinolones* | 0.90 [0.82 - 0.95] | 0.82 [0.46 - 0.93] | 0.95 [0.90 - 0.98] | 0.90 [0.80 - 0.95] | 0.96 [0.92 - 0.99] | 0.92 [0.85 - 0.97] |
| *Piperacillin- tazobactam* | 0.88 [0.79 - 0.93] | 0.78 [0.16 - 0.88] | 0.93 [0.87 - 0.97] | 0.86 [0.74 - 0.94] | 0.95 [0.89 - 0.98] | 0.90 [0.80 - 0.96] |
| *Co-trimoxazole* | 0.73 [0.57 - 0.82] | 0.57 [0.18 - 0.76] | 0.84 [0.72 - 0.91] | 0.71 [0.53 - 0.83] | 0.87 [0.77 - 0.95] | 0.77 [0.61 - 0.90] |

*Table 4s. Odds Ratio of having an antibiogram sensitive to an antibiotic regimen for groups of age with the associated 95%HDI assessing the probability with 3-5 years as reference category.*

|  | ***Overall cohort*** | ***Non-complex cohort*** |
| --- | --- | --- |
|  | *OR [95% HDIs]* | *OR [95% HDIs]* |
| *< 1 month* | 1.18 [0.68-1.78] | 1.33 [0.76-2.15] |
| *2-6 months* | 1.84 [1.18-2.69] | 2.43 [1.41-3.74] |
| *7-24 months* | 1.4 [0.93-1.91] | 1.75 [1.08-2.54] |
| *6-10 years* | 1.89 [1.26-3.21] | 2.55 [1.51-5.46] |
| *11-15 years* | 2.32 [1.18-7.08] | 2.65 [1.15-13.65] |

*Figure 1s. Monte Carlo Markov Chains (MCMC) traceplots of the WISCA model’s parameters.*


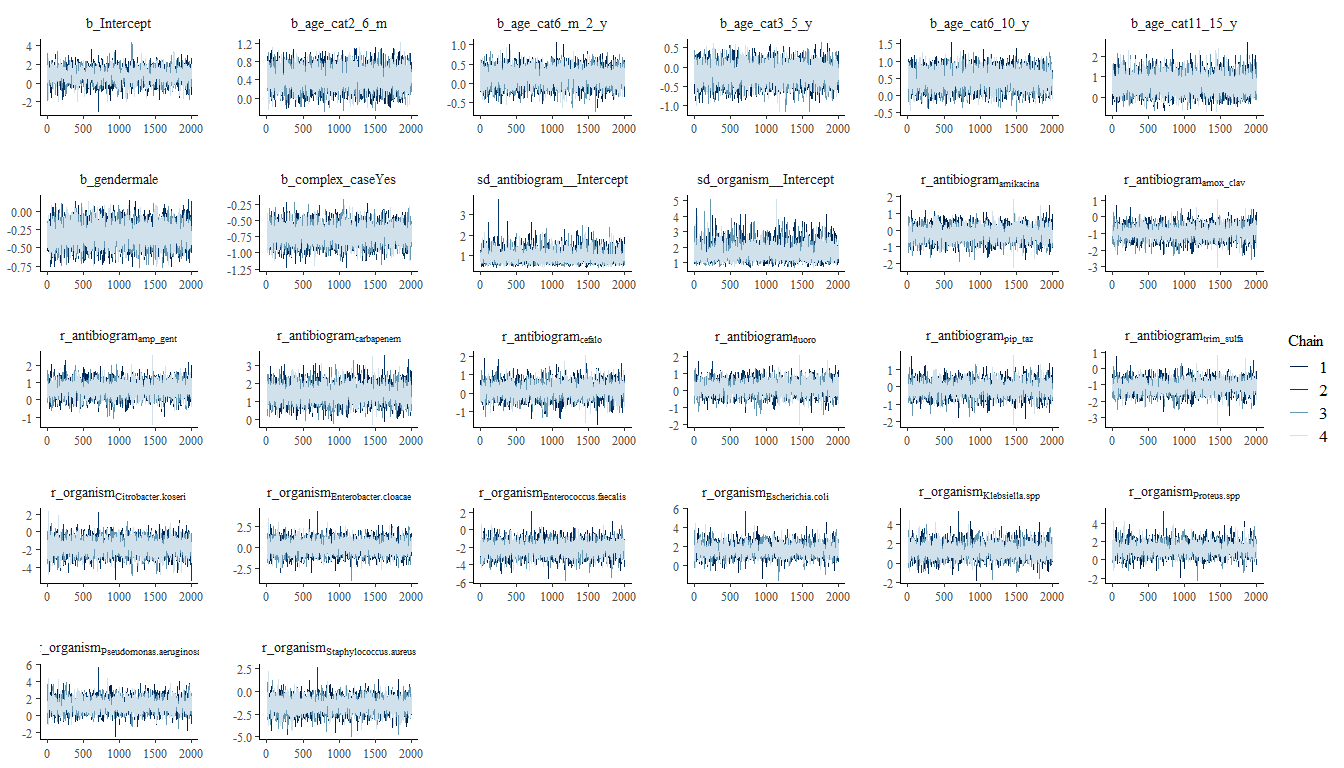


*Figure 2s. Density plots of the posterior distributions of the parameters of the model. The parameter names that start with b identify the fixed effects coefficients, whereas standard deviation and varying intercepts parameter names start with sd and r, respectively.*


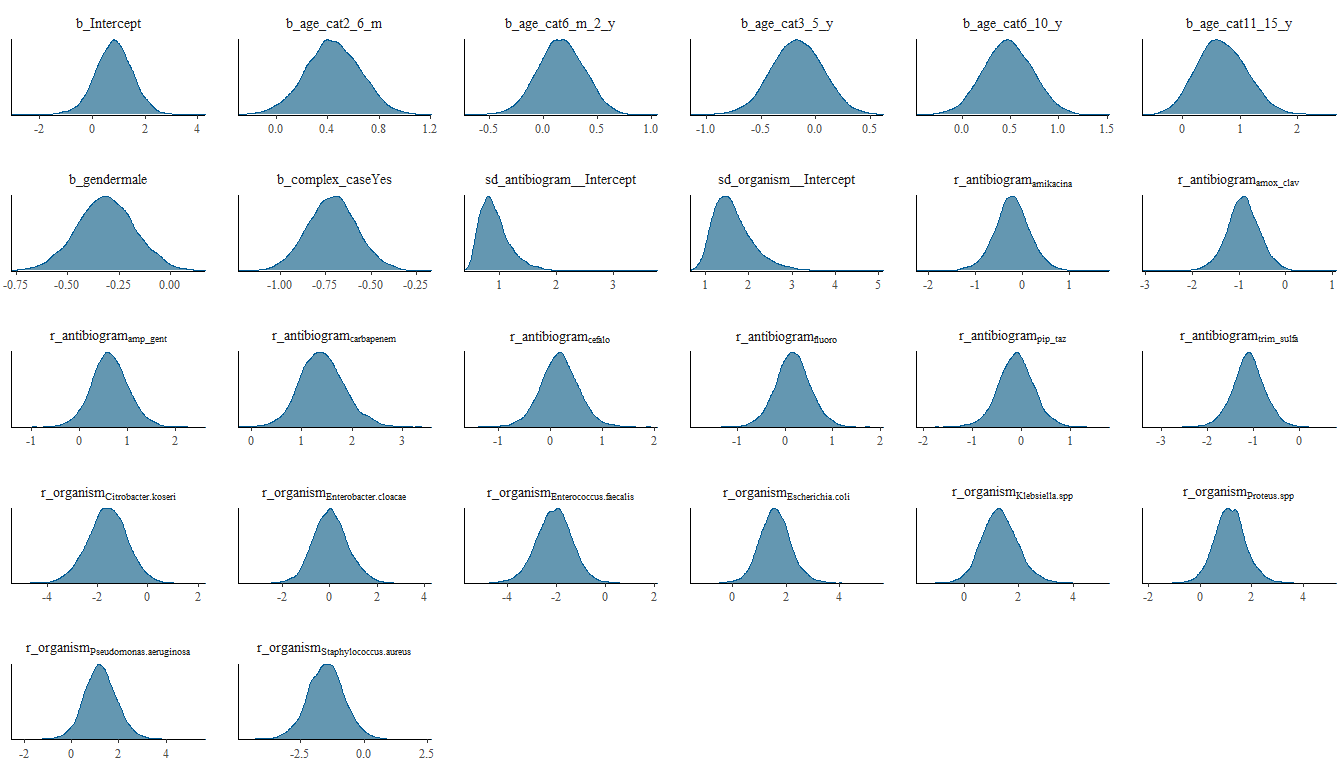


*Figure 3s. Example of a E. coli cumulative antibiogram of urine samples stratified by ward (General Pediatric) and age class (1-14 years) available at Centre B that describes susceptibility rates (expressed as percentage) for different antibiotics tested including data from January 2018 to June 2018.*


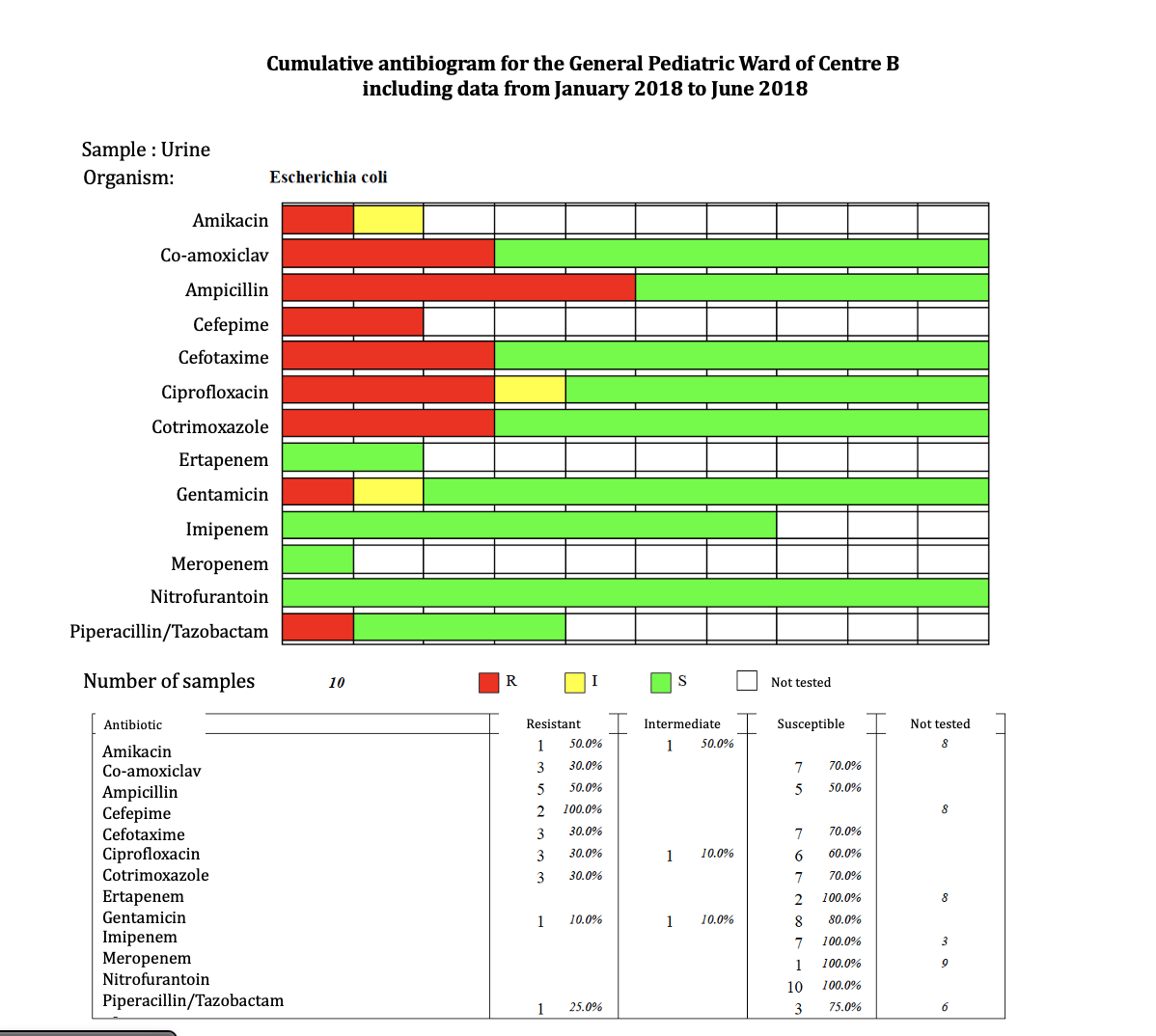

Supplement: Supplementary file 1 — Additional file 1. Supplementary Figures and Tables. [file 13756_2021_939_MOESM1_ESM.docx]
